# Supplementary figures and images for: Squalene Monooxygenase Gene SsCI80130 Regulates Sporisorium scitamineum Mating/Filamentation and Pathogenicity
Source: J Fungi (Basel). 2022 Apr 30;8(5):470. doi: 10.3390/jof8050470 (PMC9143649; doi:10.3390/jof8050470)

Figure S1

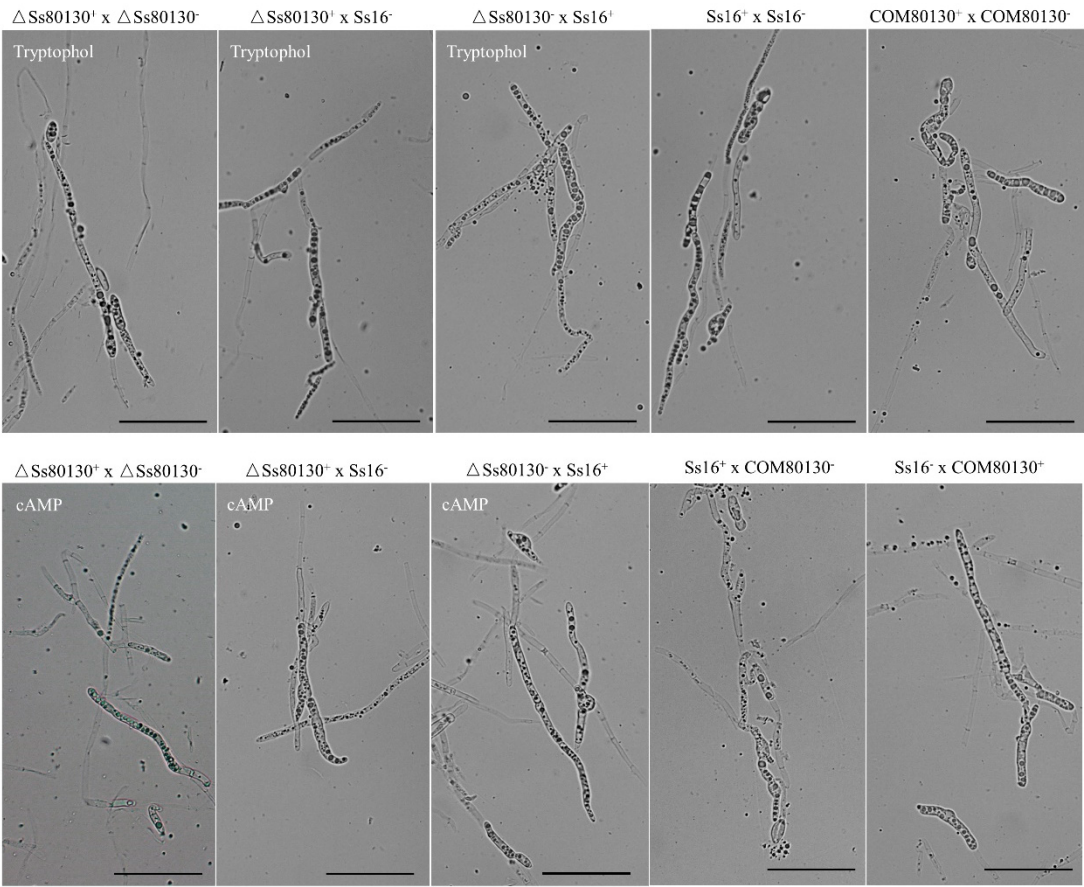

Supplement: Supplementary file 1 [file jof-08-00470-s001.zip › jof-1686666-supplementary.pdf]
